# Direct and indirect selection on mate choice during pollen competition: Effects of male and female sexual traits on offspring performance following two‐donor crosses

**DOI:** 10.1111/jeb.13684

**Published:** 2020-08-10

**Authors:** Josefin A. Madjidian, Henrik G. Smith, Stefan Andersson, Åsa Lankinen

**Affiliations:** ^1^ Biodiversity Department of Biology Lund University Lund Sweden; ^2^ Center for Environmental and Climate Research Lund University Lund Sweden; ^3^ Plant Protection Biology Swedish University of Agricultural Sciences Alnarp Sweden

**Keywords:** indirect benefit, pollen tube growth rate, resource allocation, sexual selection and conflict, stigma receptivity

## Abstract

Mate choice in plants is poorly understood, in particular its indirect genetic benefits, but also the direct benefits of avoiding harmful matings. In the herb *Collinsia heterophylla*, delayed stigma receptivity has been suggested to enhance pollen competition, potentially functioning as a female mate choice trait. Previous studies show that this trait can mitigate the cost of early fertilization caused by pollen, thus providing a direct benefit. We performed two‐donor pollinations during successive floral stages to assess how this stigma receptivity trait and two pollen traits known to affect siring success influence indirect benefits in terms of offspring performance. We also investigated differential resource allocation by studying the influence of sibling performance in the same capsule. Offspring performance in terms of flower number was mainly affected by parental identities and differential resource allocation. Offspring seed production showed some influence of resource allocation, but was also affected by pollen donor identity and varied positively with late stigma receptivity. However, the effect of late stigma receptivity on offspring seed production was weakened in matings with pollen that advanced stigma receptivity. In conclusion, delayed stigma receptivity may be selected through both direct and indirect fitness effects in *C. heterophylla*, where pollen‐based delay on stigma receptivity might act as a cue for mate choice. However, selection may also be counteracted by antagonistic selection on pollen to advance stigma receptivity. Our results highlight the challenges of studying indirect genetic benefits and other factors that influence mate choice in plants.

## Graphical abstract

A combination of pollen‐based and pistil‐based onset of stigma receptivity affects offspring seed production following the two‐donor crosses in *Collinsia heterophylla*, suggesting a joint effect of direct and indirect benefits of enhancing pollen competition.

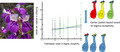

## 1 INTRODUCTION

Mate choice, a process that generates nonrandom variation in partner mating success, is common in many species and a central tenet of sexual selection (Andersson, 1994; Darwin, 1871; Hosken & House, 2011). Selection on mate choice can be caused either by indirect genetic benefits, such as when preference and the trait targeted by preference become genetically correlated and/or when attractive traits reflect broad genetic quality (Andersson, 1994; Fisher, 1958; Kokko, Jennions, & Brooks, 2006; Zahavi, 1975), or by direct benefits to females, for example nuptial gifts, high‐quality territory and parental care (Trivers, 1972) or the ability to minimize costly matings in a sexual conflict scenario (Cameron, Day, & Rowe, 2003; Kokko & Jennions, 2014; Parker, 1979). Direct and indirect effects could also act simultaneously on mate choice (Andersson & Simmons, 2006; Arnqvist & Rowe, 2005; Kokko & Jennions, 2014).

Sexual selection can occur in many organisms where sexually selective forces mainly act after gamete release, including plants, fungi, simultaneously hermaphroditic animals, and sperm and broadcast spawners (Beekman, Nieuwenhuis, Ortiz‐Barrientos, & Evans, 2016; Moore & Pannell, 2011; Skogsmyr & Lankinen, 2002). However, sexually selective forces in plants, particularly sexual conflict, remain relatively underexplored (Lankinen & Karlsson Green, 2015). Male–male competition in plants could occur, for example, over the ability to disperse pollen by wind or by insect vectors (Christopher, Mitchell, & Karron, 2019; Delph & Ashman, 2006; Tonnabel, David, & Pannell, 2019) or as pollen competition in the pistil, analogous to sperm competition in animals (Bernasconi et al., 2004). Pollen competition imposes selection among haploid pollen genotypes (Immler & Otto, 2018; Mulcahy, 1979) on traits conferring high pollen competitive ability (e.g. pollen tube growth rate, Snow & Spira, 1991; pollen germination rate, Swanson, Hammond, Carlson, Gong, & Donovan, 2016; and pollen size, McCallum & Chang, 2016). During pollen competition, there is an opportunity for female mate choice if pistil traits favour some pollen over others by enhancing pollen competition (Wilson & Burley, 1983; e.g. long style, Ramesha et al., 2011; large stigmatic area, Armbruster, 1996; and delayed stigma receptivity, Galen, Schykoff, & Plowright, 1986) or by chemical signalling in the stigma and style (Bhattacharya & Baldwin, 2012).

Enhanced pollen competition induced by females can improve mean offspring quality (Labouche, Richards, & Pannell, 2017; Marshall & Whittaker, 1989; Mulcahy, 1971; Quesada, Fuchs, & Lobo, 2001; Skogsmyr & Lankinen, 2000). However, it remains unclear whether superior offspring results from mate choice for indirect genetic benefits or from other possible indirect genetic benefits of sorting among pollen, for example increased mate compatibility or paternal diversity (Bernasconi et al., 2004; Lyons, Ware, Price, Antonovis, & Motten, 1989; Moore & Pannell, 2011; Pélabon, Albertsen, Falahati‐Anbaran, Wright, & Armbruster, 2015; Skogsmyr & Lankinen, 2002) or avoidance of pistil contact or fertilization by pollen that causes female reproductive costs, that is sexual conflict (e.g. Lankinen, Hellriegel, & Bernasconi, 2006). Another source of uncertainty concerns differences in offspring vigour caused by post‐zygotic processes, such as differential maternal provisioning of the first fertilized ovules, or ovules fertilized under high pollen loads or by genetically superior pollen (e.g. Delph, Weinig, & Sullivan, 1998; Pélabon et al., 2015).

Pollen deposition can cause changes in floral receptivity and attractiveness of many angiosperms (at least 60 genera, van Doorn, 1997), mediated by, for example, perianth senescence (wilting), changes in flower colour, termination of nectar and scent production, and development of the ovary (O’Neill, 1997; Primack, 1985). Previous work on the annual herb *Collinsia heterophylla* Buist (Plantaginaceae) demonstrated pollen‐induced sexual conflict over the timing of stigma receptivity: some pollen donors fertilize ovules when their pollen is applied to partially receptive stigmas at early floral stages at the expense of reduced seed production by the pollen recipient (Lankinen, Hydbom, & Strand, 2017; Madjidian, Hydbom, & Lankinen, 2012). Because late stigma receptivity increased maternal seed production following two‐donor pollinations during early floral development (Lankinen, Smith, Andersson, & Madjidian, 2016), it is possible that delayed stigma receptivity has been selected for by a direct benefit of avoiding early fertilizing donors. Other studies of *C. heterophylla* suggest that delayed stigma receptivity could enhance pollen competition (Lankinen & Armbruster, 2007; Lankinen & Madjidian, 2011), indicating that this trait also could provide indirect benefits. On the other hand, delayed stigma receptivity may not directly influence mate choice, as siring success in two‐donor crosses was most strongly favoured by the ability of pollen to induce late stigma receptivity rather than recipient influence on this trait (Lankinen et al., 2016). To better understand whether delayed stigma receptivity can function as a mate choice trait for indirect benefits by promoting pollen competition, it is important to investigate how measures of offspring fitness covary with the ability of the maternal and paternal parent to induce early or late stigma receptivity.

In this study, we investigated the potential for selection on mate choice during pollen competition in *C. heterophylla*, with particular focus on delayed stigma receptivity as a potential mate choice trait. Using data from our previously reported crossing experiment, analysed with respect to male siring success and maternal seed production (Lankinen et al., 2016), we evaluated offspring performance (measured by flower and seed production) in relation to pollen and pistil traits of the parents, after two‐donor pollinations performed at different stages of floral development. Because our study species *C. heterophylla* has a mixed mating system (with both selfing and outcrossing), we allowed pollen donors to compete with either outcross or self‐pollen to span the full range of possible mating patterns and thus better mimic natural conditions. We assessed two pollen traits as potential cues for mate choice*—*pollen tube growth rate (measured in vitro) and pollen‐based ability to influence stigma receptivity—based on their correlations with siring success in two‐donor crosses (Lankinen et al., 2016) and in crosses with sequential arrival of pollen (Lankinen & Strandh, 2016). We predicted (i) that pollen donor identity affects offspring performance, (ii) that this effect is at least partly linked to specific pollen traits and (iii) that late pistil‐based onset of stigma receptivity has positive effects on offspring performance. Moreover, given that late stigma receptivity enhances maternal seed production, we tested whether the benefit of late stigma receptivity carried over to the offspring generation as a direct benefit by performing a parent–offspring regression for seed production. We also explored effects of resource allocation on offspring performance.

## 2 MATERIALS AND METHODS

### 2.1 Plant material

*Collinsia heterophylla* is an annual hermaphrodite, native to the California Floristic Province, North America (Neese, 1993; Newsom, 1929). *C. heterophylla* is self‐compatible, and mean population outcrossing rates range between 0.29 and 0.84 (Kalisz et al., 2012; Strandh, Jönsson, Madjidian, Hansson, & Lankinen, 2017). Plants used in the current study originated from a natural population in Mariposa County, California (N 37.502; W 120.123), with an intermediate outcrossing rate of 0.45 (Strandh et al., 2017).

The flowers, which are arranged in whorls on spikes, have undehisced anthers and a short, undeveloped pistil with an immature, unreceptive stigma at flower opening. The four anthers dehisce sequentially during 3–4 days, during which the style elongates and the stigma matures and becomes receptive (Armbruster et al., 2002). *C. heterophylla* is not strictly protandrous (male function preceding female function), as the stigma commonly becomes receptive before all anthers have dehisced. Self‐pollination can occur autonomously late during a flower's active life stage, when the style is sufficiently long to contact the anthers, provided they still contain pollen (Armbruster et al., 2002; Kalisz et al., 1999). The pistil of each flower contains up to 20 functional ovules (based on maximum number of seeds found in a seed capsule, Madjidian & Lankinen, 2009) and develops into a dry dehiscent capsule.

We used plants derived from seeds sampled by seed family from about 50 open‐pollinated plants from the natural population. We grew plants in the greenhouse for two generations to establish a completely outbred base population and to identify individuals homozygous for the genetic marker used in the paternity analysis. The marker is manifested as a dark band on the upper corolla lip controlled by a dominant allele at a single locus; thus, plants homozygous for the recessive allele lack the band (Lankinen, 2009). We raised experimental plants from cold‐stratified seeds and grew them under pollinator‐proof conditions in a semi‐automated greenhouse for two generations during the winter and spring of 2009 (two‐donor pollinations) and 2010 (offspring generation). All plants grew in unfertilized potting compost (peat with 10% clay and 2% calcium) mixed with sand (4:1) without additional fertilizer in pots of volume 565 000 mm^3^. Greenhouse plants with this pot size are comparable to or slightly larger than plants in the field; the number of seeds per capsule is less affected by pot size than the number of flowers produced (Lankinen & Hydbom, 2017). We watered plants as needed, every other day at the seedling stage and almost every day during the adult stage. To avoid position effects on plant performance, we rotated all plants among positions on benches several times during the experiment.

### 2.2 Two‐donor pollinations

**Figure 1:**
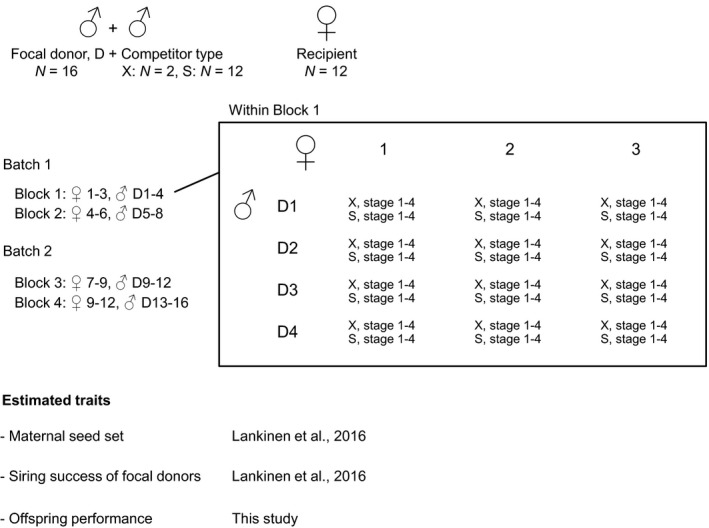
Experimental design to assess maternal seed production, siring success of focal donors (D, dark genetic marker) following competition with either two outcross standard donors (X) or self (S)‐pollen (white genetic marker) (as reported in a companion study, Lankinen et al., 2016) and its relationship with subsequent offspring performance in *Collinsia heterophylla*. Pollinations were performed at each of four floral stages. Twelve recipients (represented by four full‐sibs each) were hand‐pollinated with 16 focal donors. Each donor was crossed with three unrelated recipients and each recipient with four unrelated donors in one of four experimental blocks (only a fraction of the crosses are shown). Blocks belong to two batches separated in time by one month. See Methods for details

We analysed data on offspring performance from the same set of two‐donor pollinations as used for previous analyses of male siring success and maternal seed production (Lankinen et al., 2016) (Figure 1). We mixed an equal amount of pollen from two donors and applied the mixed pollen as one pollination, as described in Lankinen et al. (2016). We performed pollinations at each of four stages of floral development (days 1–4 after flower opening) to determine whether dependent variables vary with the stage of pollination. We collected the ripe capsules and stored them in a cold room until the second year of the experiment when we determined paternity and evaluated their performance.

Crosses involved 16 focal pollen donors homozygous for the dominant marker trait (possessing a dark band on the upper floral lip; denoted ‘dark’) that competed with a donor of the white, and recessive type (lacking the dark band; denoted ‘white’) on 12 white recipients, each consisting of four full siblings (denoted ‘recipients’) (Figure 1). Crosses performed on the four siblings per recipient were pooled together (see Lankinen et al., 2016). Focal dark donors each competed either with two white standard donors (one at a time) or with self‐pollen from the white recipient. Crosses were performed in two batches separated by one month, involving four experimental blocks (two per batch) in which each of four dark donors was crossed with each of three unrelated recipients (Figure 1). The genotypes of the two white standard donors were the same across blocks. In total, we conducted 2,304 crosses across the 12 recipients (192 crosses per recipient = four dark outcross donors per recipient x [two competing white outcross donors + one competing self‐donor] x four stages x four replicates).

The crosses generated a total of 18,274 seeds (stage 1—2,430 seeds; stage 2—4,629 seeds; stage 3—5,482 seeds; and stage 4—5,737 seeds). On average, only 4% of all crosses were unsuccessful and all parent combinations produced seeds, as expected for a species with no known self‐incompatibility system. As with previous studies (Madjidian, Hydbom, et al., 2012), the failure rate was highest for stage 1 crosses (20%). To evaluate male siring success (Lankinen et al., 2016) and offspring performance, we sowed 16 seeds per stage and recipient–donor combination and followed the resulting plants to seed production (for description of offspring performance measures, see below).

### 2.3 Pollen and pistil traits measured

As described in Lankinen et al. (2016), we recorded in vitro pollen tube growth rate and pollen‐based onset of stigma receptivity for the 16 dark pollen donors, as well as pistil‐based onset of stigma receptivity for the 12 plants serving as recipients, to be able to relate these traits to male siring success, maternal seed production and offspring performance. We determined pollen tube growth rate as the average length of ten pollen tubes per sample in a drop of Hoekstra medium (Hoekstra & Bruinsma, 1975) for 1 hours 45 min in a dark chamber at a constant temperature of 20–21°C. We measured pollen‐based onset of stigma receptivity, which represents the influence of pollen on timing of stigma receptivity (Madjidian, Andersson, & Lankinen, 2012), as the earliest floral stage during which pollen from a given donor resulted in seed production on other individuals (data averaged over three unrelated recipients); likewise, we quantified pistil‐based onset of stigma receptivity as the earliest floral stage at which flowers on a given recipient set seed with pollen from other individuals (data averaged over three unrelated donors, for details, see Lankinen et al., 2016). We derived data on pollen‐ and pistil‐based onset of stigma receptivity from a companion study conducted in parallel to the crosses in 2009 (using full‐sibs to the plants in the current study) that aimed to estimate heritability and genetic correlations (Madjidian, Andersson, et al., 2012).

### 2.4 Measurements of offspring performance

To assess the impact of pollination stage, type of cross, recipient, donor and particular pollen and pistil traits on offspring performance, we focused on offspring representing the dark morph, that is plants that must have been sired by the focal dark donor, although data from white offspring provided insights into differential resource allocation (see Statistical Procedures). We scored a maximum of six offspring per recipient‐/donor‐stage combination for two ‘late’ fitness‐related traits, the number of flowers per plant (henceforth ‘flower number’) and the number of seeds per capsule produced by autonomous selfing under pollinator‐free conditions (henceforth ‘offspring seed production’). These traits were uncorrelated (*r* = .004, *df* = 499) and have earlier been used as performance measures in the present study system (Lankinen & Armbruster, 2007; Lankinen & Madjidian, 2011). Previous data from the same population (Lankinen & Madjidian, 2011) show that number of seed capsules produced per plant in the main spike is positively correlated with both flower number per plant (*r* = .22, *df* = 480, *p* < .0001) and autonomous seed production per capsule (*r* = .11, *df* = 479, *p* < .013). Thus, there is no obvious trade‐off between number of seeds per capsule and number of seed capsules. In addition, number of seeds per capsule did not differ between selfed and outcrossed flowers in the same population (Lankinen & Strandh, 2016; Madjidian, Hydbom, et al., 2012), indicating that our measure of seed production should be representative of that in a natural population with mixed mating.

We estimated flower number by multiplying the number of branches and the number of flowers on the main spike. We estimated offspring seed production per capsule by averaging the number of seeds in three capsules per individual.

In addition to the two offspring performance traits, we also estimated their product (seed number x flower number), representing total offspring fitness. The measure of total fitness assumes equal contribution to fitness of both offspring performance traits and may be sensitive to environmental influences, especially on flower number (Lankinen & Hydbom, 2017).

### 2.5 Statistical procedures

#### 2.5.1 Mixed‐model analyses

We analysed our data on measures of offspring performance—seed production (square‐root‐transformed) and flower number, as well as the estimate of total offspring fitness (seed number x flower number)—as a function of all fixed factors, that is type of competitor pollen [self or outcross], floral stage at pollination, pollen tube growth rate, pollen‐ and pistil‐based onset of stigma receptivity and all possible two, three‐ and four‐way interactions. We standardized quantitative predictors to a mean of zero and a standard deviation of one to allow direct comparison between regression coefficients (Zuur, Ieno, & Smith, 2007). Due to a missing value for pollen tube growth rate of one pollen donor, only 15 donors were included in the analyses. The pollen traits used as predictors were uncorrelated (*p* = .87, Lankinen et al., 2016). The random structure of the model included block as well as donor, recipient and their interaction with each other and with all possible combinations with type of competitor and stage at pollination, all nested within block. In this way, we controlled for repeated measurements of parental identities. We did not include batch in the model, but assume that any batch effect should be captured by the blocking factor (batch 1 = block 1–2, batch 2 = block 3–4). Including batch as part of the random structure and nesting all other factors within batch led to poor model estimates. Moreover, batch had no significant effect on either measure of offspring performance (*p* > .52) when included as a fixed factor in the model.

We fitted the model with a linear mixed‐model approach, using restricted maximum likelihood (REML) in the package lme4 (Bates, Maechler, Bolker, Walker, & Christensen, 2018) in R (R Development Core Team, 2018). Model selection and significance testing of the final fixed‐model parameters were conducted with MCMC simulation in the R package MCMCglmm (Hadfield, 2018a), an approach chosen because it provided estimates > 0 for all variance components. We specified random factors excluding interactions with stage using a compound symmetry structure with the prior of the covariance parameter following a Cauchy distribution with a standard deviation of 25 (Gelman, 2006). We specified random factors including interactions between stage using a general covariance structure with an inverse‐Wishart prior parametrized with a diagonal variance matrix multiplied by 0.01 and a degree of belief parameter of five (Hadfield, 2018b). The variance matrix is controlling for unequal correlations between adjacent floral stages. We specified fixed factors with a noninformative prior and the residual error term with an inverse‐Wishart distribution having unit variance and a degree of belief of 0.001. We used 5,000,000 of MCMC iterations, a thinning interval of 5,000 and a burn‐in of 15,000. Model selection was conducted by removing factors one by one according to the deviance information criterion (DIC, a generalization of the AIC criterion suitable for MCMC simulations), starting from higher‐order interactions, until the best model was identified (lower DIC = higher model fit) (Hadfield, 2018b). The competitor‐by‐stage interaction was kept in all models. Estimated marginal means were calculated from the model using the R package emmeans (Lenth, 2018).

We evaluated effects of the random factors specified above, and their interactions with fixed factors, by inspecting their relative contribution to the residual variance and comparing DIC values for the full model with removal of interactions. To test when a random factor had a significant contribution, we used REML and LR tests in the R package lmertest (Kuznetsova, Brockhoff, & Christensen, 2018).

#### 2.5.2 Analyses of inter‐trait associations

As a complementary approach, we used linear regression based on donor‐ or recipient‐specific means to quantify the causal effect of pollen and pistil traits on offspring performance, measured by seed and flower number, as well as the estimate of total offspring fitness (seed number x flower number). Due to the missing value for pollen tube growth rate in one pollen donor, only an univariate regression was performed for pollen traits. We also regressed mean offspring seed production on mean recipient seed production (the latter based on data from the experimental hand pollinations) to determine whether the benefit of late stigma receptivity (increased seed production) can be carried over to the offspring generation as a direct genetic benefit of mate choice. To account for possible environmental differences between generations, we standardized both offspring and recipient measures to a mean of zero and a standard deviation of one (resulting in an intercept equal to 0). Since flower number was only assessed in offspring, we were unable to test for any recipient–offspring association in this trait.

#### 2.5.3 Allocation effects

We assessed the possible contribution of differential allocation of maternal resources between fruits by investigating how seed production or flower number of white outcross offspring covaried with that of focal dark offspring from the same capsule. If the performance of white offspring (sired by the same two competing white donors) is positively correlated with the performance of their dark siblings (sired by one of 16 different focal dark donors), the differences in white offspring performance between crossings can be at least partly attributed to differential allocation of resources between capsules. We used seed production or flower number in white offspring as the dependent variable, respectively, and tested the fixed effects of seed production or flower number in dark offspring from the same crossing combination and developmental stage, as well as the random effects of block, and recipient, donor and their interaction with each other and with stage (all nested within block). We fitted linear mixed models in lme4 (using REML) and performed model selection and significance testing of the final fixed‐model parameters with MCMC simulation, as described above.

## 3 RESULTS

### 3.1 Impacts of pollen donor and recipient identity on offspring performance

Identity of the focal dark pollen donor contributed relatively more to the variation in offspring seed production than the recipient identity and the recipient‐by‐donor interaction (posterior mean relative to residual variation: 0.15 versus 0.03 and 0.02, respectively, Table 1). As shown by the DIC values, the model fit was improved by including the highest order interaction. For offspring flower number, both donor and recipient identity, but not their interaction, explained the largest part of the variation (posterior mean relative to residual variation: 0.23 and 0.27 versus 0.03, respectively, Table 1), suggesting a stronger maternal influence than that observed for offspring seed production. Based on the DIC values, all three‐way interactions improved the model fit; the highest order interaction added little to the explanatory power of the model.

**Table 1:** Posterior mean and lower and upper 95% CI (credible interval) of factors included in the random structure of the mixed model evaluated with MCMC simulation for seeds per capsule and flower number in offspring from two‐donor pollinations in *Collinsia heterophylla*

Measure/Factor	Posterior mean	Lower 95% CI		Upper 95% CI	Posterior mean factor/residual
Seeds per capsule DIC full model: 739.27 versus removing four‐way interaction: 741.73					
Block	0.0652	<0.0001		0.0685	0.33
Recipient, Rec (Block)	0.0061	<0.0001		0.0243	0.03
Donor (Block)	0.0297	<0.0001		0.0814	0.15
Rec × Donor (Block)	0.0042	<0.0001		0.0167	0.02
Rec × Stage (Block)	−0.0003–0.0190				<0.10
Donor × Stage (Block)	0.0017–0.0216				<0.11
Rec × Competitor type, Comp (Block)	0.0040	<0.0001		0.0149	0.02
Donor × Comp (Block)	0.0085	<0.0001		0.0302	0.04
Rec × Donor × Stage (Block)	−0.0008–0.0220				<0.11
Rec× Donor × Comp (Block)	0.0027	<0.0001		0.0102	0.01
Rec × Stage × Comp (Block)	−0.0005–0.0208				<0.11
Donor × Stage × Comp (Block)	−0.0130–0.0329				<0.17
Rec × Donor × Stage × Comp (Block)	−0.0013–0.0228				<0.12
Residual	0.194	0.166		0.227	
Flower number DIC full model: 11,437.19 versus. removing interactions, four‐way: 11,436.29 or any three‐way: >11,437.36					
Block (Batch)	575		0.01	2,360	0.08
Recipient, Rec (Block)	1,860		308	4,040	0.27
Donor (Block)	1,560		265	3,160	0.23
Rec × Donor (Block)	229		0.0003	659	0.03
Rec × Stage (Block)	−0.31–0.64				<0.01
Donor × Stage (Block)	−0.01–0.47				<0.01
Rec × Competitor type, Comp (Block)	97		0.001	321	0.01
Donor × Comp (Block)	60		0.0006	236	0.01
Rec × Donor × Stage (Block)	−0.70–2.6				<0.01
Rec × Donor × Comp (Block)	302		0.0006	679	0.04
Rec × Stage × Comp (Block)	−0.57–1.9				<0.01
Donor × Stage × Comp (Block)	−14–13				<0.01
Rec × Donor × Stage × Comp (Block)	−6.6–19				<0.01
Residual	6,850		6,200	7,500	

Note

Model selection was based on DIC (deviance information criterion). Stage refers to the floral stage at which the recipient flower was pollinated. Competitor type refers to the type of competitor pollen (self or outcross). Random factors are nested under block. The posterior mean for interactions involving stage is reported as a range of the 16 values resulting from interactions between each of the four stages. Posterior mean factor/residual represents the proportional importance of the factor relative to the residual variation.

Testing the significance of random factors in LR tests showed results similar to comparing the relative contribution to variance in the MCMC simulation: offspring seed production was significantly affected by donor identity (χ^2^ = 5.50, *df* = 1, *p* = .019) and the interaction between donor, competitor type and stage (χ^2^ = 6.32, *df* = 1, *p* = .012). Offspring flower number was most strongly influenced by recipient (χ^2^ = 13.1, *df* = 1, *p* < .001), but also by donor (χ^2^ = 8.92, *df* = 1, *p* = .003) and by the four‐way interaction (χ^2^ = 5.45, *df* = 1, *p* = .020). No other factors were statistically significant.

### 3.2 Pollen and pistil traits affect offspring seed production but not flower number

**Figure 2:**
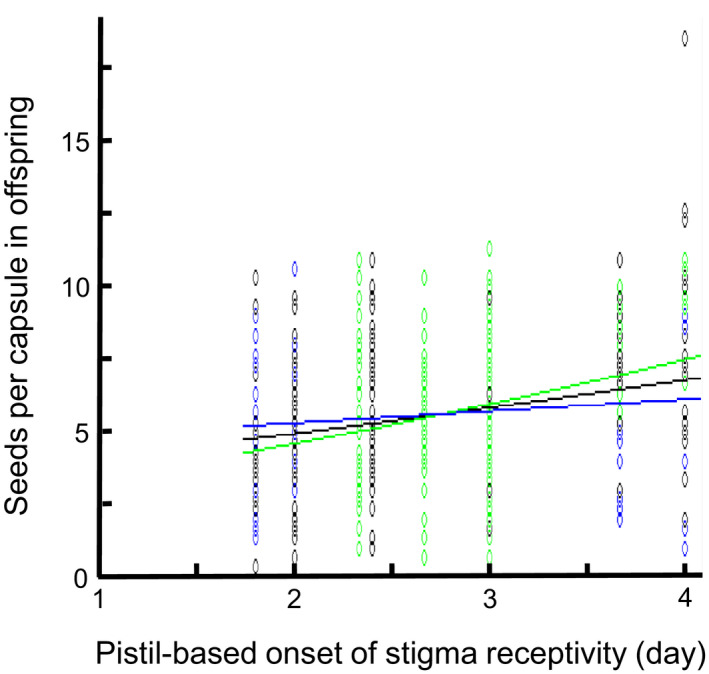
Seeds per capsule in offspring were influenced by an interaction between maternal pistil‐based onset of stigma receptivity and paternal pollen‐based onset of stigma receptivity in *Collinsia heterophylla* following mixed‐model analysis with MCMC simulation. The data and relationship were back‐transformed in the graph from square‐root‐transformed seeds per capsule (measured as mean of three autonomously selfed capsules per recipient), and pistil‐ and pollen‐based onset standardized to a mean of 0 and a standard deviation of 1 (using mean = 2.76, SD = 0.658 versus. mean = 2.74, SD = 0.344). The effect of pistil‐based onset was only evident when fathered by donors with late pollen‐based onset (as exemplified by the green line, standardized pollen‐based onset = 1, y = 2.37 + 0.195x). For earlier pollen‐based onset, the relationship was weakened (black line, standardized pollen‐based onset = 0, y = 2.37 + 0.126x; blue line, standardized pollen‐based onset = −1, y = 2.37 + 0.056x). Green circles = pollen‐based onset > 2.91, black circles = pollen‐based onset 2.57 < *i* < 2.91, blue circles = pollen‐based onset < 2.57. *N* recipients = 12, *N* donors = 15

**Figure 3:**
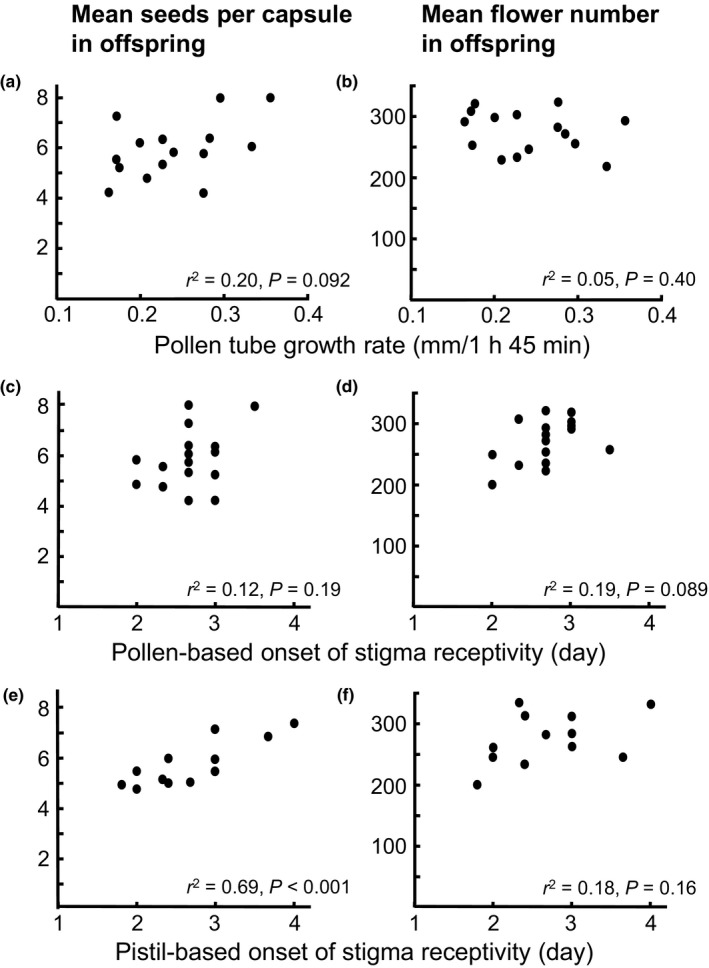
Offspring performance in relation to measures of pollen and pistil traits of parents. Offspring performance was averaged over self‐ and outcross competitors and four floral stages at pollination, measured as (a), (c), (e) mean seeds per capsule (square‐root‐transformed, mean of three autonomously selfed capsules per recipient) and (b), (d), (f) mean flower number (estimated as number of branches × number of flowers in main spike) in *Collinsia heterophylla*. Seeds per capsule were back‐transformed in the graph. r2 = squared correlation coefficient

MCMC simulation of the mixed models showed no statistically significant effects of floral developmental stage at pollination or competitor type for the two measures of offspring performance (Table 2, Fig. S1). For offspring seed production, there was a statistically significant interaction between pistil‐ and pollen‐based onset of stigma receptivity (Table 2), reflecting a positive synergistic effect of late pistil‐based and late pollen‐based onset (Figure 2). Pistil‐based onset considered over all combinations of pollen‐based onset was statistically nonsignificant with a *P*‐value of 0.052 (Table 2). Pollen tube growth rate also showed a statistically nonsignificant positive influence (*p* = .076, Table 2). The regression analysis based on recipient means similarly suggested a positive relationship between pistil‐based onset of stigma receptivity and offspring seed production (*F*_1,10_ = 22.5, *p* < .001, Figure 3e), although the effects of both pollen traits failed to reach statistical significance (pollen tube growth rate: *F*_1,13_ = 3.30, *p* = .092; pollen‐based onset: *F*_1,14_ = 1.92, *p* = .19) (Figure 3a,c). Thus, although both analyses confirmed a positive influence of late pistil‐based onset on offspring seed production, this effect was weakened for offspring fathered by pollen donors with early pollen‐based onset (Figure 2).

**Table 2:** Parameter estimates and upper and lower 95% CI (credible interval) of predictors after mixed‐model analysis with MCMC simulation, assessing effects of pollen and pistil traits, type of competitor pollen (self or outcross) and floral stage at pollination on seeds per capsule, flower number and their combined value in offspring from two‐donor pollinations in *Collinsia heterophylla*

Measure/Factor	Estimate	Lower 95% CI	Upper 95% CI	*p*
Seeds per capsule DIC full model: 714.50 versus removing pollen‐ and pistil‐based onset interaction: 716.28				
Intercept	**2.35**	**2.01**	**2.70**	**<.001**
Competitor type self, Comp S	0.118	−0.188	0.388	.38
Stage 2	−0.006	−0.295	0.274	.98
Stage 3	−0.029	−0.309	0.219	.84
Stage 4	0.048	−0.226	0.299	.77
Comp S × Stage 2	−0.218	−0.625	0.158	.25
Comp S × Stage 3	−0.057	−0.380	0.302	.72
Comp S × Stage 4	−0.077	−0.456	0.261	.69
Pollen tube growth, PTG	0.110	−0.021	0.236	.076
Pollen‐based onset, PO	0.003	−0.124	0.147	.96
Pistil‐based onset, PI	0.126	−0.001	0.258	.052
PO × PI	**0.070**	**0.003**	**0.143**	**.030**
Flower number DIC full model: 11,033.26 versus. removing pistil‐based onset: 11,038.94				
Intercept	**277**	**234**	**318**	**<.001**
Competitor type self, Comp S	22.1	−3.84	49.2	.10
Stage 2	−6.29	−24.4	14.1	.48
Stage 3	−6.64	−26.4	10.3	.50
Stage 4	2.77	−15.9	21.7	.72
Comp S × Stage 2	−5.73	−37.5	25.3	.74
Comp S × Stage 3	0.378	−31.0	30.9	1.00
Comp S × Stage 4	−14.5	−47.3	16.0	.37
Pistil‐based onset, PI	21.0	−10.4	53.5	.17
Seeds per capsule × flower number DIC full model: 6,689.745 versus. removing any of two four‐way interactions: >6,690.044				
Intercept	**672**	**586**	**751**	**<.001**
Competitor type self, Comp S	23.5	−67.0	138	.65
Stage 2	−40.0	−115	31.4	.30
Stage 3	−31.1	−109	40.4	.41
Stage 4	−8.57	−87.0	63.3	.86
Comp S × Stage 2	−46.5	−179	78.7	.47
Comp S × Stage 3	−6.87	−139	125	.90
Comp S × Stage 4	29.5	−101	164	.67
Pollen tube growth, PTG	2.64	−76.6	74.6	.95
Pollen‐based onset, PO	−76.1	−149	78.7	.064
Pistil‐based onset, PI	**119**	**41.1**	**209**	**.004**
Comp S × PTG	19.5	−88.8	120	.67
Comp S × PO	55.9	−38.7	182	.34
Comp S × PI	−104	−222	5.31	.066
Stage 2 × PTG	10.9	−60.2	100	.81
Stage 3 × PTG	−32.8	−110	47.2	.49
Stage 4 × PTG	64.0	−11.8	150	.14
Stage 2 × PO	**82.6**	**7.71**	**171**	**.046**
Stage 3 × PO	**102**	**16.1**	**193**	**.022**
Stage 4 × PO	**95.4**	**8.60**	**181**	**.026**
Stage 2 × PI	−61.2	−151	25.9	.18
Stage 3 × PI	−40.4	−135	41.7	.38
Stage 4 × PI	**−95.8**	**−186**	**−11.1**	**.024**
PTG × PO	−0.027	−103	109	.99
PTG × PI	39.1	−25.4	102	.23
PO × PI	22.1	−55.3	99.1	.56
Comp S × PTG ×PO	−102	−202	12.8	.062
Comp S × PTG ×PI	14.7	−43.5	74.2	.65
Comp S × PO ×PI	−28.0	−131	74.7	.59
Stage 2 × PTG ×PO	−6.69	−117	81.6	.90
Stage 3 × PTG ×PO	−34.1	−143	59.1	.52
Stage 4 × PTG ×PO	23.2	−78.9	131	.70
Stage 2 × PTG ×PI	**−74.0**	**−140**	**−5.83**	**.042**
Stage 3 × PTG ×PI	−42.4	−117	29.1	.26
Stage 4 × PTG ×PI	−20.4	−92.2	41.5	.56
Stage 2 × PO ×PI	−18.0	−98.8	61.7	.67
Stage 3 × PO ×PI	−47.1	−131	41.4	.30
Stage 4 × PO ×PI	−0.746	−82.8	86.5	.96
Comp S × Stage 2 × PTG	7.77	−136	136	.90
Comp S × Stage 3 × PTG	72.4	−61.1	197	.27
Comp S × Stage 4 × PTG	−67.4	−204	69.0	.30
Comp S × Stage 2 × PO	−55.2	−209	79.4	.45
Comp S × Stage 3 × PO	−54.4	−197	93.3	.47
Comp S × Stage 4 × PO	−86.1	−237	42.7	.22
Comp S × Stage 2 × PI	101.4	−20.8	233	.14
Comp S × Stage 3 × PI	107.6	−31.9	237	.12
Comp S × Stage 4 × PI	**167**	**32.4**	**312**	**.016**
PTG × PO ×PI	15.3	−53.1	84.5	.68
Comp S × Stage 2 × PO ×PI	85.7	−33.5	211	.18
Comp S × Stage 3 × PO ×PI	88.3	−55.4	199	.16
Comp S × Stage 4 × PO ×PI	−52.3	−179	83.9	.45
Comp S × PSL ×PO × PI	71.3	−25.7	168	.15

Note

Model selection was based on DIC (deviance information criterion). Estimates represent the intercept = the mean for the reference level outcross competitor and stage 1, and partial regression coefficients = the means for the remaining levels expressed as differences from the reference mean. Significant parameter estimates are presented in bold.

For offspring flower number, all pollen traits were removed during model selection in the MCMC simulation (Table 2). Accordingly, no detectable statistically significant effects of pollen or pistil traits were found in any analysis (Table 2; regression analyses based on recipient means: pollen tube growth rate: *F*_1,13_ = 0.756, *p* = .40; pollen‐based onset: *F*_1,14_ = 3.33, *p* = .089, pistil‐based onset: *F*_1,10_ = 2.27, *p* = .16, Figure 3b,d,f).

Combining both performance traits into a single fitness measure (seed number x flower number) showed a similar positive effect for pistil‐based stigma receptivity as observed for seed number, but additional interaction terms with competitor type and stage were also retained in this model (Table 2). Pairwise comparisons between different combinations of stage and competitor type showed that the slope between pistil‐based onset and offspring fitness was significantly more positive for stage 1 compared to stage 4 in pollinations with an outcross competitor (estimate = 95.3, highest posterior density interval = 11.1–186). No other pairwise comparisons were statistically significant. For pollen traits, pollen tube growth rate had little influence on offspring fitness, whereas the effect of pollen‐based onset varied across stages (Table 2). Pairwise comparisons indicated a statistically significant difference between a negative slope in stage 1 and a positive slope in stage 3 (estimate = −74.3, highest posterior density interval range = −154‐(−8.09)). The regression analyses on the combined offspring measure over all stages and competitor types suggested statistically significant positive effects for both pistil‐based stigma receptivity (*F*_1,10_ = 10.4, *r^2^* = 0.51, *p* = .009) and pollen‐based onset (*F*_1,14_ = 6.11, *r^2^* = 0.30, *p* = .027). No such effect was seen for pollen tube growth rate (*F*_1,13_ = 0.193, *r^2^* = 0.015, *p* = .67).

### 3.3 Positive correlation between recipient and offspring seed production

In line with a genetically heritable effect on seed production, there was a significantly positive statistical relationship between the mean recipient seed production (based on hand‐outcrossed flowers) and the mean seed production of their respective offspring (based on autonomously pollinated flowers) (y = 0.821x, *F*_1,10_ = 20.7, *p* = .001).

### 3.4 Resource allocation affects offspring performance

**Figure 4:**
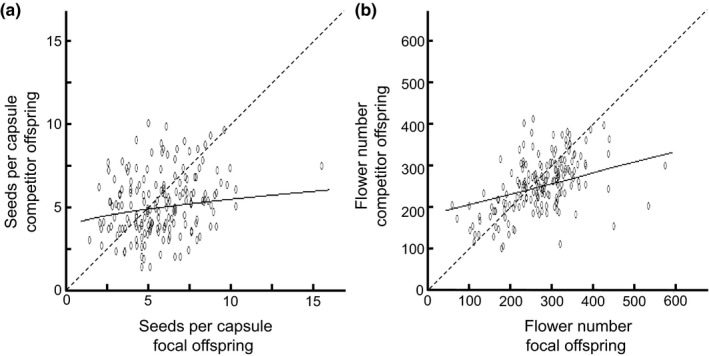
Performance of offspring fathered by focal dark pollen donors covaried with performance of offspring fathered by outcross competitor white donors within the same seed capsule in *Collinsia heterophylla* following mixed‐model analyses with MCMC simulation, indicating an effect of maternal resource allocation between seed capsules. The relationships for (a) seeds per capsule (square‐root‐transformed, measured as mean of three autonomously selfed capsules per recipient): y = 2.22 + 0.061x, and for (b) flower number (estimated as number of branches × number of flowers in main spike): y = 282 + 21.0x. Performance of focal offspring was standardized to a mean of 0 and a standard deviation of 1. The data and relationships were back‐transformed in the graph (using mean = 2.37, sd = 0.43 for seed production and mean = 247, sd = 20.8 for flower number of focal offspring). The dashed line represents a 1:1 relationship

As many as 92.6% of the evaluated recipient–donor combinations (each based on 16 offspring per stage of pollination) produced offspring sired by both of the two competing pollen donors (the focal one with dark marker and the competitor with white marker). Thus, there was ample opportunity to assess the influence of differential allocation of resources between capsules by comparing seed production or flower number of outcross offspring from the same recipient but having different donors (one dark and one white). Although both seed production and flower number covaried positively between siblings sired by the dark and white donor (Figure 4), the relationship was more strongly statistically significant for offspring flower number (*p* < .001) than for offspring seed production (*p* = .038) (Table 3).

**Table 3:** Posterior mean/parameter estimates and upper and lower 95% CI (credible interval) of random factors and predictors after mixed‐model analysis with MCMC simulation, for effects of maternal resource allocation between seed capsules evaluated by the influence of performance of focal offspring and floral stage at pollination on competitor outcross offspring performance in *Collinsia heterophylla*

Measure/factor		Model estimates			
Seeds per capsule in competitor outcross offspring DIC full model: 118.20 versus. removing focal offspring seeds per capsule: 121.62					
	Factor	Estimate	Lower 95% CI	Upper 95% CI	*p*
Fixed	Intercept	2.19	1.55	2.99	.002
	Stage 2	0.044	−0.134	0.232	.63
	Stage 3	0.089	−0.099	0.278	.33
	Stage 4	0.002	−0.193	0.187	.98
	Focal offspring seeds per capsule	**0.061**	**0.005**	**0.119**	**.038**

	Factor	Posterior mean	Lower 95% CI	Upper 95% CI	Posterior mean factor/residual
Random	Block	1.46	0.001	2.05	17.4
	Recipient, Rec (Block)	0.0072	<0.0001	0.028	0.09
	Dark donor (Block)	0.0050	<0.0001	0.028	0.06
	Rec × Dark donor (Block)	0.0098	<0.0001	0.027	0.12
	Rec × Stage (Block)	−0.0016–0.264			<3.1
	Dark donor × Stage (Block)	−0.0013–0.018			<0.21
	Residual	0.084	0.062	0.105	

Flower number in competitor outcross offspring DIC full model: 1918.167 versus. removing focal offspring flower number: 1927.498					
	Factor	Estimate	Lower 95% CI	Upper 95% CI	*p*
Fixed	Intercept	**246**	**210**	**284**	**<.001**
	Stage 2	−5.70	−26.3	13.4	.56
	Stage 3	0.87	−18.2	20.7	.95
	Stage 4	9.11	−8.99	27.7	.34
	Focal offspring flower number	**20.8**	**11.4**	**28.9**	**<.001**

	Factor	Posterior mean	Lower 95% CI	Upper 95% CI	Posterior mean factor/residual
Random	Block	1920	0.0013	3,250	0.90
	Recipient, Rec (Block)	1,030	100	2,430	0.48
	Dark donor (Block)	164	0.0001	556	0.08
	Rec × Dark donor (Block)	244	<0.0001	663	0.11
	Rec × Stage (Block)	−0.008–0.27			<0.01
	Dark donor × Stage (Block)	−0.023–0.25			<0.01
	Residual	2,130	1,680	2,670	

Note

Model selection of fixed factors was based on DIC (deviance information criterion). Random factors are nested under block. The posterior mean for interactions involving stage is reported as a range of the 16 values resulting from interactions between each of the four stages. Estimates represent the intercept = the mean for the reference level stage 1, and partial regression coefficients = the means for the remaining levels expressed as differences from the reference mean. Significant factors (*p* < .05) are presented in bold.

## 4 DISCUSSION

Using a large crossing experiment with *C. heterophylla*, we explored the function of delayed stigma receptivity in relation to multiple pollen cues for mate choice under a selective regime of indirect genetic benefits. Cross type (self versus. outcross) had no effects on offspring traits and will thus not be considered further. Offspring flower number was mainly affected by parental identities and differential allocation of maternal resources. Offspring seed production was associated with the identity of the pollen donor and timing of stigma receptivity in the maternal individual. However, the positive effect of late stigma receptivity was weakened for offspring sired by pollen donors with early pollen‐based influence on stigma receptivity. Although late stigma receptivity could be favoured by selection to provide indirect benefits in terms of fecundity, the adaptive significance of pollen traits as cues for indirect benefits is uncertain and may be counteracted by antagonistic selection on pollen to advance stigma receptivity.

### 4.1 Pollen traits acting as cues for indirect benefits of mate choice

In animals, indirect benefits of mate choice have been extensively assessed (Andersson, 1994; Hosken & House, 2011). The few plant studies that assessed indirect benefits of pollen competitive traits showed positive effects of rapid pollen tube growth on offspring performance in both *Betula pendula* (Pasonen, Pulkkinen, & Käpylä, 2001) and *Viola tricolor* (Skogsmyr & Lankinen, 2000), whereas no such effects could be found for pollen germination rate in *Silene latifolia* (Jolivet & Bernasconi, 2007). Here, we found a significant effect of donor identity on both flower number and seed production in offspring of *C. heterophylla*. In a recent review, 37 of 56 studies detected an influence of the paternal parent on seed germination (Baskin & Baskin, 2019), but these studies rarely followed offspring beyond seed traits. The paternal influence observed in *C. heterophylla* suggests that it is favourable for maternal plants to prefer certain donors over others.

To explore the possibility for pollen traits to function as a cue for mate choice, we focused on fast pollen tube growth rate and late pollen‐based onset of stigma receptivity, two traits previously shown to be correlated to siring success in *C heterophylla* (Lankinen et al., 2016; Lankinen & Strandh, 2016). Since our previous analyses of the crosses analysed in the current study did not include block in the error structure and used a multimodel approach (Lankinen et al., 2016), we reanalysed our previous data on male siring success using our current approach to make the results directly comparable between studies. The reanalysis confirmed that late pollen‐based onset rather than fast pollen tube growth rate or pistil‐based onset was most important for high siring success, particularly when competing with self‐pollen at early stages compared to late stages (pairwise comparison of slope in stages 1 and 4: estimate = 0.154, highest posterior density interval = 0.0013–0.317). In the present study, pollen tube growth rate and offspring seed production were positively, though not significantly, correlated. It is thus unlikely that this trait could function as a reliable cue for mate choice. Late pollen‐based onset of stigma receptivity was connected with increased offspring seed production when mated with recipients with late pistil‐based onset of stigma receptivity, that is recipients expected to enhance pollen competition the most; notably, the positive effect of this pollen trait remained significant in regression analysis using the estimate of total offspring fitness (seed number x flower number) as a dependent variable. Thus, late pollen‐based onset of stigma receptivity could conceivably act as a more reliable pollen cue for providing indirect benefits of female mate choice, at least under conditions involving simultaneous arrival of two pollen donors.

An important limitation of the present study is that our offspring performance traits may be weakly correlated with lifetime reproductive success (Walsh & Blows, 2009) due to overriding influences of, for example, differences in mating ability (Andersson, 1994), and that all results concern plants grown under benign greenhouse conditions. For example, natural populations may be under strong selection for early flowering (Elle, Gillespie, Guindre‐Parker, & Parachnowitsch, 2010; Kudo, 2006), a factor that would reduce (or outweigh) the importance of overall flower and seed number as fitness components.

### 4.2 Delayed stigma receptivity and indirect versus. direct selection on mate choice

Although studies of animals have documented a positive genetic correlation between female preference and offspring fitness (e.g. Hine, Lachish, Higgie, & Blows, 2002), the few such studies in plants have largely been theoretical (Lankinen & Skogsmyr, 2001; Mulcahy, 1983). Our present results from *C. heterophylla*, showing a synergistic effect of late pollen‐ and pistil‐based onset of stigma receptivity on offspring seed production, together with previous detection of significant heritable variation in the ability to delay stigma receptivity (Madjidian, Andersson, et al., 2012), suggest that this pistil trait has evolutionary potential. The positive effect of late stigma receptivity was also seen for the combined fitness measure, although its magnitude depended on stage and competitor type. An alternative explanation for the link between late stigma receptivity and increased offspring seed production is that fully receptive pistils are better at enhancing competition among pollen independent of pollen donor, thus favouring pollen grains of superior genetic quality within pollen loads representing single donors (Mulcahy, 1979; Walsh & Charlesworth, 1992). This mechanism will not favour some pollen donors over others and is therefore conceptually unrelated to sexual selection. However, the significant effect of pollen donor on offspring seed production in our crosses involving constant pollen load size is not consistent with the latter, alternative hypothesis.

Despite evidence from *C. heterophylla* that delayed stigma receptivity can enhance pollen competition (Lankinen & Armbruster, 2007; Lankinen & Madjidian, 2011), there is some uncertainty regarding the influence of pistil‐based onset on male siring success (Lankinen et al., 2016; see also reanalysis above), a weakness of the mate choice scenario involving indirect benefits. However, pistil‐based onset was correlated with mean siring ability across stages (*r^2^* = 0.38, *df* = 12, *p* = .032, Pearson correlation), potentially suggesting that delayed stigma receptivity could have some influence on which pollen donors are favoured over others. It is also possible that the pistil can control paternity in alternative ways, for example, by allowing pollen to grow in multiple waves through the stylar tissue (Losada & Herrero, 2017) or by direct protein–protein interactions (Guo, Halitschke, Wielsch, Gase, & Baldwin, 2019). In future studies, more detailed mechanistic studies of pollen competing in the pistil are needed (cf. Harder, Aizen, Richards, Joseph, & Busch, 2016; Lora, Hormaza, & Herrero, 2016).

The positive effect of late stigma receptivity on both recipient (Lankinen et al., 2016) and offspring seed production could also reflect direct benefits, which usually are believed to be a stronger selective force than indirect benefits during the evolution of mate preference (Cameron et al., 2003; Kirkpatrick & Barton, 1997). Our finding of a significant positive parent–offspring relationship for seed production, in conjunction with a direct benefit of delaying stigma receptivity in the maternal generation in particular at early compared to intermediate stages (Lankinen et al., 2016; confirmed here by reanalysis: pairwise comparison of slope in stage 1 and 2: estimate = 0.254, highest posterior density interval = 0.0583–0.425), indeed indicates that the benefits of late stigma receptivity can be expressed in the offspring generation as a side effect of high heritability. Because another study found a negative correlation between pistil‐ and pollen‐based stigma receptivity in the same plant individual (Hersh et al., 2015), it does not appear likely that the positive effect on seed production in matings between recipients with delayed stigma receptivity and donors with late pollen‐based receptivity was caused only by genetic correlations. Interestingly, early pollen‐based influence on stigma receptivity had a negative effect on offspring seed production in recipients with late stigma receptivity, but not in recipients with early stigma receptivity. This could potentially lead to selection for direct benefits by the loss of mate choice or mate choice for early fertilizing pollen, thereby avoiding costs of the sexual conflict over stigma receptivity (cf. Li & Holman, 2018). In previous crosses at early floral stages in *C. heterophylla*, in which pollen competitors arrived sequentially, early rather than late stigma receptivity was related to the highest maternal seed production (Lankinen & Strandh, 2016). Thus, the relative benefits of direct versus. indirect mate choice may be different under alternative pollen arrival schedules. Additional studies are needed to better understand the role of direct versus. indirect mate choice in *C. heterophylla*.

### 4.3 Influence of differential resource allocation

Analogous to the difficulty of assessing cryptic mate choice in animals (Andersson & Simmons, 2006), mate choice in plants may be confounded by post‐zygotic mechanisms such as preferential allocation of resources to the first fertilized ovules or ovules fertilized under high pollen loads (e.g. Delph et al., 1998). To evaluate the effects of differential resource allocation or other maternal effects in the present study, we compared performance of white outcross offspring to that of dark offspring from the same capsule. Both performance traits measured on white offspring covaried positively with the same trait measured on dark focal offspring, despite all white offspring being fathered by the same two standard competitors. Thus, we conclude that these offspring traits are affected by the overall resource level available to the fruit during seed development. Offspring flower number appeared more strongly affected by resources from the maternal parent compared to offspring seed production, a difference also seen in a soil resource manipulation experiment (Lankinen & Hydbom, 2017). The covariation between dark and white offspring is consistent with increased maternal allocation to particular fruits, for example those with high‐quality seeds acting as strong resource sinks (Ida, Harder, & Kudo, 2013; Pélabon et al., 2015), although direct measurements of seed or fruit mass will be needed to fully evaluate this hypothesis. Other mechanisms are, however, also possible. For example, increased pollen competition caused by higher competitive ability of a superior dark donor could favour increased competition for resources between seeds sired by different pollen donors, leading to increased offspring fitness (Marshall & Ellstrand, 1986). Although our results highlight the importance of controlling for resource allocation in studies of indirect mate choice, it remains to be seen whether pollen traits cause differential resource allocation.

### 4.4 Conclusions

Although previous studies have found a positive link between enhanced pollen competition and high offspring fitness (e.g. Mulcahy, 1971; Quesada et al., 2001), we know little about the underlying mechanisms, particularly regarding the influence of sexual selection. To our knowledge, the present experiment with *C. heterophylla*, described here and in our companion paper (Lankinen et al., 2016), is the first to test predictions regarding the evolution of mate choice in plants, sexual antagonism and differential resource allocation by simultaneously assessing the influence of male and female sexual traits on measures of male siring success, female seed production (direct benefit) and offspring performance (indirect benefit). Our companion study provides evidence that delayed stigma receptivity could evolve under a selective regime of sexual antagonism by avoiding direct costs of early fertilizing pollen (reduced maternal seed production). Although the results of the current study showed a strong influence of maternal resource allocation on offspring flower number, they also point to an indirect benefit of delaying stigma receptivity in terms of increased offspring seed production, although simple heritability effects cannot be excluded. Thus, delayed stigma receptivity could be selected in response to both direct and indirect benefits. Despite a clear effect of pollen donor on offspring seed production, it is more uncertain to what extent the investigated pollen traits could function as cues for mate choice, or if selection of pollen traits is opposing the interest from the recipient plant in a sexual conflict scenario (Lankinen et al., 2017). The multiple selective forces related to the enhancement of pollen competition highlight the challenges to understand mate choice in plants and other organisms in which sexual selection and other selection forces interact to influence mating patterns (Alonzo & Servedio, 2019; Beekman et al., 2016).

## CONFLICT OF INTEREST

The authors report no conflicts of interest.

### Peer Review

The peer review history for this article is available at https://publons.com/publon/10.1111/jeb.13684.

## Supporting information

Fig S1
