# Supplementary material for: Direct and indirect selection on mate choice during pollen competition: Effects of male and female sexual traits on offspring performance following two‐donor crosses
Source: J Evol Biol. 2020 Aug 10;33(10):1452–67. doi: 10.1111/jeb.13684 (PMC7589368; doi:10.1111/jeb.13684)
Supplement: Supplementary file 1 — Fig S1 [file JEB-33-1452-s001.docx]

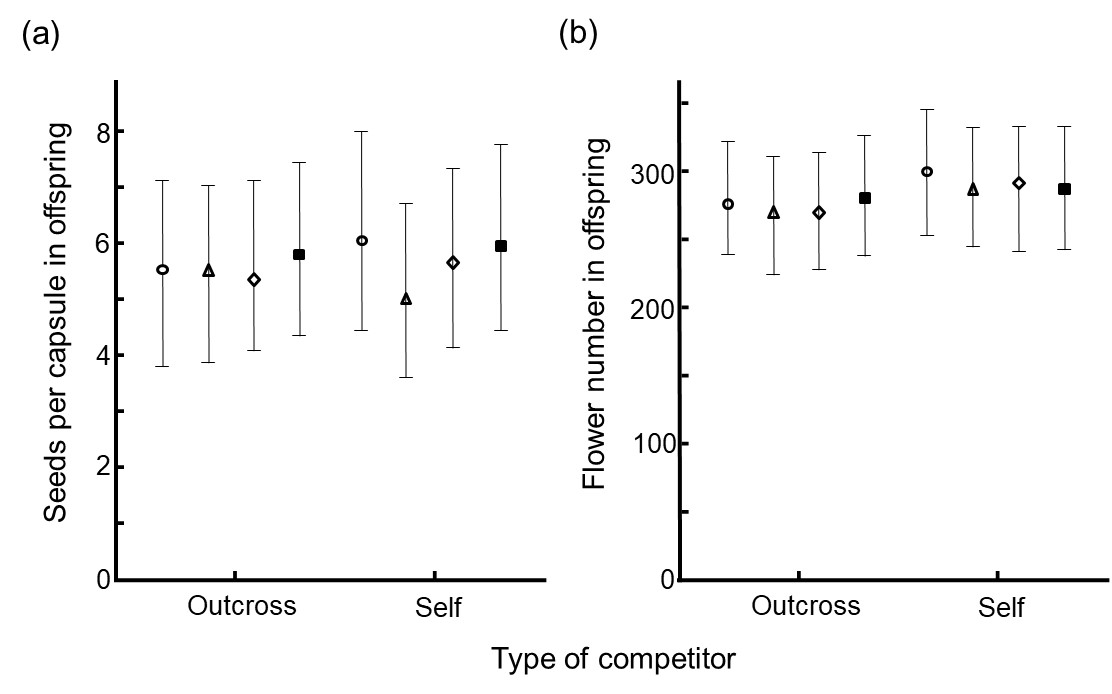


**FIGURE S1** Estimated marginal means following mixed-model analyses with MCMC simulation of performance in outcrossed offspring sired by a focal pollen donor that had either competed with pollen from an outcross or self donor at four floral stages in two-donor pollinations of *Collinsia heterophylla*. Performance was measured as (a) square-root transformed seeds per capsule (mean of three autonomously selfed capsules per recipient) and (b) flower number (estimated as number of branches × number of flowers in main spike). Seeds per capsule was back-transformed in the graph. Circles = stage 1, tringles = stage 2, diamonds = stage 3, squares = stage 4. Error bars denote 95% HPD (highest posterior density) interval probability. No differences between means were significant.
